# Evolution, Ecology and Management of Wild Boar and Deer

**DOI:** 10.3390/ani14182741

**Published:** 2024-09-22

**Authors:** Javier Pérez-González

**Affiliations:** Biology and Ethology Unit, Veterinary Faculty, University of Extremadura, 10003 Caceres, Spain; jpergon@unex.es; Tel.: +34-927-251-371

Wild boar (*Sus scrofa*) is the most widespread member of the order Artiodactyla, a group of even-toed ungulates that are prone to overabundance, with adverse consequences for conservation, agriculture, transportation and public health. This group includes several members of the family Cervidae, such as red deer (*Cervus elaphus*), roe deer (*Capreolus capreolus*), white-tailed deer (*Odocoileus virginianus*) and mule deer (*Odocoileus hemionus*). Understanding the evolution, ecology and management of wild boar and deer can help managers of these populations determine when and where a population goes from being an opportunity to a threat.

Wild boar and deer have significant impacts on natural communities and human activities worldwide [1,2,3,4]. Even-toed ungulates make up about half of the combined mass of terrestrial wild mammals, with white-tailed deer and wild boar accounting for the species with the greatest biomasses within this group [5]. Moreover, mule deer, moose (*Alces alces*), red deer and roe deer are among the eight terrestrial wild mammals with the highest biomasses globally [5]. Wild boar can significantly affect both vegetation and animal communities because they adapt to a wide range of temperatures, consume a wide variety of foods and produce many offspring [6,7]. Likewise, deer that consume a broad diet of forbs and woody browse can have a profound impact on forests [8]. Human–wildlife conflicts are exacerbated by a local overabundance of wild boar, red deer and white-tailed deer [9], as this overabundance can lead to crop damage, traffic accidents, the transmission of infectious diseases to livestock and humans [1,10] and direct conflict with people when these animal populations become habituated to human presence [11].

Large productive populations of wild boar and deer provide a valuable harvest for hunters. In addition to providing game meat and venison, which are increasingly consumed in developed countries [12], recreational hunting offers significant economic benefits, particularly for rural areas [13]. Hunting generates revenue for private landowners, conservation agencies, governments and local communities [13,14]. Moreover, the provision of services to recreational hunters supports broader local and national economies [13,15].

While concerns and management strategies primarily focus on the impacts of overabundance, wild boar and deer populations can also face challenges regarding their conservation. High-density populations of wild boar and deer are at risk of genetic introgression, which threatens their conservation by altering genetic composition [16,17]. Some deer species face conservation issues due to low population sizes [18,19]; even populations of typically abundant species are experiencing declines and raising conservation concerns [20,21]. In addition to promoting conservation, studying the evolution, ecology and management of these populations can enhance our understanding of their underlying biological processes and help us develop strategies to control them. 

This Special Issue presents recent findings on the Evolution, Ecology and Management of Wild Boar and Deer through six research articles and one review. Schütz et al. [22] explored the utility of automated computer vision techniques for detecting wild boar in camera trap images. Their study shows high precision in detecting not only wild boar but also deer and other species. Rosenberger et al. [23] investigated the indirect effects of antlered deer hunting on female white-tailed deer movements using GPS-collared individuals. They concluded that the low hunting pressure in their study area did not significantly impact female movements. Galapero et al. [24] examined tuberculosis lesions in wild boar in the context of coinfections. They found that animals previously vaccinated against Porcine Circovirus type 2 exhibited less severe tuberculosis lesions. Edge et al. [25] employed GPS collars to estimate the annual survival and fecundity of white-tailed deer categorized into various age classes. Their population growth models suggest a decline under current conditions. Pérez-González et al. [26] compared the accuracy of microsatellite and SNP markers in measuring the distribution of genetic diversity in red deer populations. While some genetic parameters quantified with both markers were correlated, SNPs offered greater precision in inferring genetic structure and multi-locus heterozygosity among red deer. Vedel et al. [27] assessed variations in carbon and nitrogen isotopic ratios in red deer populations with varying levels of intrasexual competition. They found that the nitrogen isotopic ratio in feces was lower in populations with higher intrasexual competition, indicating differing protein usage depending on the competitive context. Lastly, the review by Newman and D’Angelo [28] integrates physiological and ecological perspectives on the visual system of cervids, highlighting the interplay between their visual adaptations and ecological interactions.

The articles included in this Special Issue underscore the importance of population monitoring for both evolutionary and ecological research, as well as for effective management. Techniques such as camera trap imaging [22], GPS technology [23,25], analysis of infectious disease-related lesions [24], genetic structure assessments [26] and analysis of stable isotopes [27] can be employed to quantify variables essential for studying species biology and monitoring populations. This Special Issue discusses perspectives on wild boar and deer as overabundant species causing human–wildlife conflicts [22,24,26]. However, two articles focus on the opposite situation, where human activities [23] and local increases in predator populations can negatively impact deer species, leading to local population declines [25]. Additionally, some contributions highlight the crucial role of basic research in understanding the evolution, ecology and behavior of these species, which in turn provides relevant information for conservation and management [27,28]. 

The articles in this Special Issue provide insights into future research directions focusing on wild boar and deer species. Developing and refining effective monitoring techniques will be crucial for studying and managing populations. New basic and applicable research on the health, behavior, physiology and ecology of these species could help mitigate human–wildlife conflicts while also addressing the conservation challenges faced by wild boar and deer populations. Future studies should also delve into the ongoing debate about the relevance of genetic analysis (especially that conducted using neutral markers [29]; see [30]) in wildlife management [31]. Furthermore, the use of vaccinations could increase the effectiveness of wildlife management and reduce the impacts of infectious diseases [24]. However, such artificial interventions might introduce new evolutionary processes, necessitating careful consideration of their long-term effects [32]. Future research should examine these impacts and identify the conditions under which vaccination is appropriate. 

Recreational hunting is considered the most effective tool for addressing threats of wild boar and deer populations and harnessing the opportunities they provide [33]. However, a decline in the number of hunters has been reported in developed countries, and the growth of wild boar and deer populations is expected to continue. In the future, it will be essential to conduct studies addressing this decline. Additionally, integrating biological and ethical perspectives may be crucial for developing alternative, effective methods for population control, particularly for wild boar.
